# WELL-BEING AND SERVICE USE AMONG VETERANS’ CAREGIVER-SURVIVORS

**DOI:** 10.1093/geroni/igae098.1095

**Published:** 2024-12-31

**Authors:** Erin Bouldin, Richard Munoz, Ranak Trivedi, Luci Leykum, Jared Hansen, Stuti Dang

**Affiliations:** University of Utah, Salt Lake City, Utah, United States; South Florida Veteran Affairs Foundation for Research and Education, Miami, Florida, United States; Stanford University/VA Palo Alto Healthcare System, Palo Alto, California, United States; South Texas Veterans Health Care System, Austin, Texas, United States; VA Salt Lake City Healthcare System, Salt Lake City, Utah, United States; Miami VA Healthcare System, Miami, Florida, United States

## Abstract

Transitioning from the role of caregiver to survivor following the death of the care recipient can be difficult. Our goal was to describe well-being and bereavement-related service use among family caregivers of Veterans who had recently died. During June-November, 2023, we sent surveys to caregivers of 1,468 Veterans who had participated in a previous survey and whose Veterans had died 3-18 months prior to this survey; 296 (20.1%) caregiver-survivors completed the survey. We assessed caregiver-survivors’ anxiety (Generalized Anxiety Disorder-2) and depressive (Patient Health Questionnaire-2) symptoms along with physical and mental health-related quality of life (HRQOL; PROMIS 10 mental and physical health) at the time of the survey. We assessed their use of specific support services since the Veteran’s death. 14% of caregiver-survivors screened positive for anxiety and 15% for depressive symptoms. Most had poorer physical HRQOL (67% had t-scores>50) and mental HRQOL (85%) than the general population. Service use was uncommon: 10% of caregiver-survivors had used a bereavement support group, 7% had used counseling/therapy, and 2% each had used a caregiver support group or VA bereavement services. 20% of caregiver-survivors said they were not aware of bereavement/counseling services but would have liked to use them. In this sample of caregiver-survivors, anxiety and depressive symptom prevalence was similar to the general population but physical and mental HRQOL were substantially poorer. Most caregiver-survivors did not use any formal services following the Veteran’s death. While most non-users were not interested in services, some reported they would have used them if offered.

